# The Impact of a Dried Fruit and Vegetable Supplement and Fiber Rich Shake on Gut and Health Parameters in Female Healthcare Workers: A Placebo-Controlled, Double-Blind, Randomized Clinical Trial

**DOI:** 10.3390/microorganisms9040843

**Published:** 2021-04-14

**Authors:** Marie van der Merwe, Damien Moore, Jessica L. Hill, Faith H. Keating, Randal K. Buddington, Richard J. Bloomer, Anyou Wang, Dale D. Bowman

**Affiliations:** 1 Center for Nutraceuticals and Dietary Supplement Research, College of Health Sciences, University of Memphis, 106 Elma Roane Fieldhouse, Zach Curlin Rd., Memphis, TN 38152, USA; dcmoore3@olemiss.edu (D.M.); Jessica.Lynn.Hill@colostate.edu (J.L.H.); fhkating@gmail.com (F.H.K.); rkb.btf@gmail.com (R.K.B.); rbloomer@memphis.edu (R.J.B.); 2 Harry Feinstone Center for Genomic Research, University of Memphis, Memphis, TN 38152, USA; awang1@memphis.edu; 3 Department of Mathematical Sciences, University of Memphis, Memphis, TN 38152, USA; ddbowman@memphis.edu

**Keywords:** dried fruit and vegetable supplement, polyphenols, fiber, microbiome, glucose metabolism

## Abstract

*Aim:* Phytochemicals from fruits and vegetables are known to reduce inflammation and improve overall health. The objective of this study was to determine the effect of a fruit and vegetable concentrate (FVC) and high fiber component on the gut microbiome in an overweight/obese, female population. *Methods:* The study was a randomized, double blind, placebo-controlled trial with 57 asymptomatic, pre-menopausal, overweight/obese females between 25–50 years of age working in healthcare. Blood and fecal samples were collected before and after two, four and five months of daily supplementation. Metabolic parameters were measured, and the gut microbiome analyzed. *Results:* No effect was observed with FVC supplementation for blood lipids, glucose and immune parameters. There was an improvement in glucose clearance. The FVC supplement did not result in taxonomic alterations at phyla level, or changes in α or β diversity, but reduced Bacteroides abundance and increased fecal butyrate. An additional high fiber component improved levels of health associated bacteria. *Conclusion:* The results suggest that a dried fruit and vegetable supplement, with a high fiber meal replacement can alter the intestinal microbiota and improve glucose clearance, suggesting that this combination of supplements can improve glucose metabolism and possibly reduce the risk of insulin resistance.

## 1. Introduction

Epidemiological studies have long demonstrated a strong association between increased fruit and vegetable consumption and a decreased risk for the development of chronic diseases such as cardiovascular disease and type 2 diabetes (T2DM) [1,2,3] Many of these diseases are driven by overnutrition-induced obesity where the adipose tissue hypertrophy results in increased proinflammatory molecules, impaired insulin signaling and ultimately dysregulation of glucose and lipid metabolism [4]. In addition, obesity is characterized by altered gut microbiome and loss of microbial diversity [5,6].

The health promoting properties of plants go beyond the provision of basic micro-and macronutrients as they also contain phytochemicals that function as antioxidants, phytoestrogens and anti-inflammatory agents [1]. Over the last decade it has become clear that plant components including polyphenols and microbiota-accessible carbohydrates (MACs) found in fruits and vegetables shape the composition and associated functions of the gut microbiota. Plant-rich diets have specifically been shown to expand gut-resident microbial populations that are beneficial to human physiology and overall health by contributing to critical host functions including nutrient [7]—and drug metabolism [8], maintenance of the mucosal barrier [9], immunomodulation [10] and protection against pathogens [11]. Plant polyphenols have been shown to alter the gut microbiome; polyphenols from blueberries can increase beneficial *Bifidobacterium spp.* [12], while cranberry extract inhibits Clostridiales in high fat/high sucrose-fed mice [13]. Polyphenols from plum prevent weight gain in obese rats with corresponding changes in gut microbiome, including increase densities of *Faecalibacterium spp*. *Lactobacillus spp*. and *Bacteroidetes spp*., and decreased fecal short chain fatty acids [14]. In addition to polyphenols, MACs) such as inulin, also selectively increase the health promoting bacteria *Bifidobacterium* and *Faecalibacterium prausnitzii* while causing a decrease in the bile-resistant *Bilophila* [15,16]. As the bidirectional interactions between host and microbes are fully integrated into human health, a disruption of the gut ecological equilibrium, also called dysbiosis, results in increasing risk of host inflammatory conditions [17,18,19].

A dried fruit and vegetable dietary supplement (JuicePlus+^®^, The Juice Plus+ Company, Collierville, TN, USA) containing a blend of polyphenols from various plants was reported to decrease lipid peroxidation, reduce systemic inflammatory molecules in healthy volunteers, and decrease markers of protein/lipid oxidation [20]. More recently, this dried fruit and vegetable supplement was shown to reduce total cholesterol, low-density lipoprotein (LDL) and systemic TNF-α levels in an overweight, aged population [21]. The present study recruited overweight female healthcare professionals to evaluate the influences of a dried fruit and vegetable supplement (JuicePlus+^®^) on the gut microbiome and clinical indicators of lipid and glucose metabolism and systemic inflammation. An additional pilot experiment used a cohort of the supplemented group to evaluate the response to a high fiber meal replacement smoothie (Complete^®^, The Juice Plus+ Company, Collierville, TN, USA) in combination with the fruit and vegetable supplement.

## 2. Materials and Methods

### 2.1. Experimental Design

This was a randomized placebo-controlled, double blinded study. A total of 67 asymptomatic women with a BMI between 25 and 40 kg/m^2^ were enrolled in this study. Participants were required to be pre-menopausal; 25–50 years of age; working in the healthcare field; not currently smoking; have no diagnosis of metabolic or intestinal disease; limit fruit and vegetable intake to no more than 3 servings per day; not using medications or nutritional supplements that affect gut health or immunity, and not be pregnant or lactating. Subjects were randomly assigned to receive a dried fruit and vegetable concentrate supplement (FVC, JuicePlus^+^ Orchard, Garden and Berry Blends, N = 27) or placebo (microcrystalline cellulose; N = 30) capsules for 16 weeks (Arm 1). At 16 weeks the FVC group was used for a follow-up study of an additional 4 weeks to evaluate responses to consuming a meal-replacement shake (Complete^®^, *n* = 14) as the first meal of the day or continue a habitual breakfast (HB, *n* = 12) (Arm 2). Blood and fecal samples were collected at the start of the intervention (baseline) and at two and four months for all subjects after initiation of the study period and at five months for the Complete/HB groups. The study was conducted according to the guidelines laid down in the Declaration of Helsinki, and all procedures involving human subjects were approved by the Institutional Review Board of the University of Memphis. The trial was registered at www.clinicaltrials.gov (identifier no. NCT02512107).

Recruitment and sample collection occurred between January 2015 and October 2018. 114 female healthcare professionals were screened and 67 were enrolled. All subjects provided written informed consent prior to participation. Ten participants discontinued the study after enrollment, but prior to data collection. One participant in the active group finished the first arm but did not continue with the Complete^®^ meal replacement arm. All subjects were required to take a urine pregnancy test to confirm that they were not pregnant. Baseline body composition for each subject was determined using dual-energy X-ray absorptiometry (DXA) performed by a licensed technician. Health history, medication and dietary supplement usage, and anthropometric measures were obtained at baseline.

### 2.2. Study Supplement

The FVC capsules contained blended fruit, vegetable and berry juice powder concentrate derived from the following: acerola cherry, apple, bilberry, blackberry, black currant, blueberry, beetroot, broccoli, cabbage, carrot, concord grape, cranberry, elderberry, kale, orange, peach, papaya, parsley, pineapple, raspberry, red currant, spinach and tomato (Juice Plus^+®^). The FVC capsules provided 7.5 mg β-carotene, 200 mg vitamin C, 60 mg RRR-a-tocopherol and 600 mg folate. Participants randomized to the placebo group (N = 30) received identically appearing capsules containing microcrystalline cellulose. The intervention began between days 1 and 5 post-menstrual cycle to control for hormone-induced change across the menstrual cycle. Subjects were instructed to take three capsules twice daily with a meal for a total of six capsules per day (in agreement with the label use instructions for the commercial product) for 16 weeks. At the end of 16 weeks, the subjects in the FVC group (N = 27) continued for another 4 weeks with half of the cohort continuing with their habitual breakfast, while the second half consumed a meal replacement smoothie (Complete^®^) that contained 13 g of plant-based protein and 8 g of soluble and insoluble fiber, including plant cellulose, fructooligosaccharide, soy fiber, rice bran pectin and apple fiber. Participants consuming the Complete^®^ meal replacement shake were instructed to blend a ¼ cup of powder with 1 cup of skim milk, fruit juice, or a non-dairy beverage, in agreement with the label use instructions for the commercial product and use it as a meal replacement for the first meal of the day.

### 2.3. Laboratory Test Visits and Sample Collection

Participants were instructed to maintain their habitual diet and lifestyle during the study period. Food and drink intake were recorded for 7 days prior to every laboratory visit and nutritional analyses were performed at baseline, and 4 and 5 months (Table A1 and Table A2).

Fecal samples were self-collected by participants during the 24 h prior to the laboratory visit. Collected fecal samples were immediately placed in an insulated bag containing freezer packs until delivery to the laboratory, at which time the samples were stored at −80 °C. Participants reported to the laboratory in the morning after an overnight fast and blood was collected by venipuncture in serum separator and EDTA containing tubes. Plasma and serum were harvested and stored at −80 °C for batch biochemical analysis. An oral glucose tolerance test (OGTT) was performed at baseline and at 4 and 5-month visits as follows; immediately after the initial blood draw, participants consumed a 10 oz beverage containing 75 g of dextrose (Trutol^®^). Ninety minutes later another blood sample was collected via venipuncture for glucose measurement. Glucose clearance was determined by the difference (∆) in glucose concentrations between fasting glucose levels and the glucose concentration measured at 90 min.

### 2.4. Blood Chemistry and Biochemical Analysis

Fasting blood glucose and lipid (cholesterol, HDL, LDL-c, Triglyceride) levels were measured from plasma using a clinical chemical analyzer (Alfa Wasserman Vet Axcel Chemistry analyzer, Caldwell, NJ, USA). Commercially available kits were used for the measurement of estradiol (#11-ESTHU-E01, Alpco, Salem, NH, USA), oxidized LDL (#10-1143-01, Mercodia, Winston Salem, NC, USA) and zonulin in serum (#30-ZONSHI-E01 and feces (#30-ZONHU-E01, Alpco, Salem, NH, USA) following manufacturer’s instructions. Plasma cytokine and adipokine levels were measured using a magnetic bead assay (Millipore, Burlington, MA, USA) and a LUMINEX Magpix analyzer with xPONENT software for data analysis.

### 2.5. Short Chain Fatty Acid Analysis

Fecal short chain fatty acids (SCFA) were measured by ion chromatography according to Pourcyrous et al. [22]. Briefly, approximately 1 mg of the fecal sample were suspended in cold 0.001% H_2_SO_4_ at a concentration of 1 mg/mL and homogenized by sonication. Samples were further diluted (1:10) in 0.001% H_2_SO_4_, centrifuged at 14,000× *g* for 5 min to sediment suspended solids, and the supernatants were filtered through a 0.2 um filter. Acetate, formate, propionate, butyrate and isobutyrate were detected using a Dionex AS15 ion exchange column incorporated into a Dionex 600 system. Potassium hydroxide (KOH) was used as the eluent with a stepwise increase in gradient starting with 9 mM increasing over 30 min to 20 mM. The peak heights were measured and the concentration of SCFA calculated by comparing with a SCFA standard and expressed as micromole per gram of stool.

### 2.6. Fecal Collection and Sample Preparation for Microbiome Analysis

For microbiome analysis genomic DNA was extracted using the ZR Fecal DNA MicroPrep^®^ kit (ZYMO Research, Irvine, CA, USA) according to the manufacturer’s instructions. Briefly, DNA was extracted by mechanical perturbation using a bead-beater and a lysis solution to lyse bacterial cells. The suspension was centrifuged through spin column filters. The DNA bound to the filters was washed, and an additional elution resulted in twice-filtered DNA. DNA quantity and quality were determined by the absorbance ratio at 260/280 nm. Library generation and 16s rRNA sequencing were performed at the Heflin Center for Genomic Science (University of Alabama, Birmingham) according to Kumar et al. [23]. Amplicon libraries were prepared by PCR amplification of the V4 region of the 16S rRNA gene. PCR products were electrophoresed in a 1.0% agarose/Tris-borate-EDTA gel and then excised and purified using Qiagen QIAquick Gel Extraction Kit (Qiagen, cat # 28704) according to the manufacturer’s instructions. The PCR products were sequenced using NextGen sequencing Illumina MiSeq platform.

### 2.7. Microbiome Data Analysis

FASTQ conversion of the raw data files was performed following de-multiplexing using MiSeq reporter and quality assessment of the FASTQ files was done using FASTQC. Quality filtering was performed using the FASTX toolkit. Quantitative Insight into Microbial Ecology (QIIME) suite, version 1.8, was used for the reminder of the analysis as described in Kumar et al. [24]; chimeric sequences were filtered using the “identify_chimeric_seqs.py” module of USEARCH and sequences were grouped into operational taxonomic units (OTUs) using the clustering program UCLUST at a similarity threshold of 97%. The Ribosomal Database Program (RDP) classifier was trained using the Greengenes (v13.8) 16S rRNA database and provided taxonomic assignments for all OTUs at a confidence threshold of 80% (0.8). Processed sequencing data were imported into Calypso 8.84 for further analysis and data visualization [25]. Mixed effect linear regression and analysis of variance (ANOVA) were applied to determine differences between groups. The Shannon index was used to quantify inter sample variability (α-diversity). Between sample variation (β-diversity) was quantified using redundancy analysis. LEfse (linear discriminant analysis Effects Size) was applied to test for significance and perform high-dimensional biomarker identification [26].

### 2.8. Statistical Analysis

For clinical parameters, a test of normality was conducted for all control and treatment data sets using the Shapiro–Wilks test. Data sets that were not normally distributed were compared by the non-parametric Wilcoxon test, otherwise an unpaired *t*-tests were used to detect treatment effects. Data are presented as mean ± SD and statistical significance was established at *p* < 0.05.

An approach based on generalized estimating equations (GEE) was also implemented for analysis of FVC vs. Placebo. A regression model with auto-regressive working correlation matrix was assumed for the data. The mean of each test was found as: 𝜇 = 𝛽0 + 𝛽1𝐺𝑟𝑜𝑢𝑝 + 𝛽2𝑇𝑖𝑚𝑒 + 𝛽3 (𝐺𝑟𝑜𝑢𝑝 × 𝑇𝑖𝑚𝑒) where the variable Group was an indicator variable for whether the observation was in the FVC (Group = 1) or Placebo (Group = 0); Time has values 1, 2, or 3 depending on whether the measurement was for the 1st (baseline), 2nd (month 2), or 3rd (month 4) sample and Group × Time is the interaction between the two. If the estimate of 𝛽1 is significant, this was considered indicative of a significant effect of the group on the mean response. If 𝛽1 is positive, then the treatment sample mean is greater than the control sample mean and vice versa when 𝛽1 is negative. For 𝛽2, a significant estimate indicates that the means are significantly affected by the time. A positive 𝛽2 value indicates that the mean increases over time and a negative value indicates a decrease. A significant estimate of coefficient 𝛽3 is indicates there was a significant interaction between group and time and further interpretations need to account for this interaction. GEE results are given in Table A3, Appendix A).

## 3. Results

Fifty-seven participants completed the first arm of the study (FVC vs. Placebo) and 26 out of 27 finished the second arm (Shake vs. habitual breakfast (HB)). Participants at baseline had a mean BMI of 30.55 ± 4.72 kg/m^2^ and age of 36.18 ± 8.2 years, with no significant differences between the FVC and Placebo groups for age, estradiol levels, weight, BMI, body fat %, blood cholesterol, triglycerides, and fasting glucose at baseline sample collection (Table 1).

### 3.1. Oral Glucose Tolerance Test

**Figure 1:**
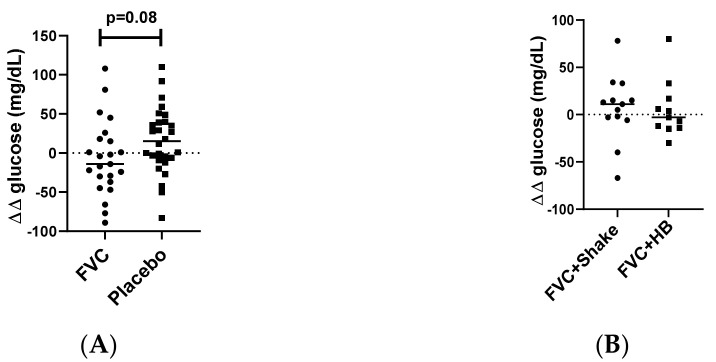
Oral glucose tolerance test. Blood glucose levels were measured after an overnight fast (x) and again 90 min after consuming a beverage containing 75 g of dextrose (y) to determine glucose clearance (∆ = y − x). The test was repeated at baseline and months 4 and 5. The difference in clearance (∆∆) between (**A**) baseline and month 4 for FVC vs. Placebo (N = 27, FVC; N = 30, placebo) and (**B**) month 4 and 5 for Shake vs. HB (*n* = 14, FVC + Shake; *n* = 12; FVC + HB) was calculated. Significance between groups were determined using non-parametric Wilcoxon test. FVC, dried fruit and vegetable supplement; HB, habitual diet.

An oral glucose tolerance test was performed at baseline and months 4 and 5 to determine changes in glucose clearance. Glucose clearance tended to be improved (value below zero, where zero is clearance equal to baseline) in the FVC group after 4 months of supplementation (*p* = 0.08, Figure 1A). GEE analysis revealed significant group (*p* = 0.017) and time differences (*p* = 0.021), though the groupxtime interaction only tended to significance (*p* = 0.094, Table A3, Appendix A). No difference was detected after the consumption of the meal replacement shake for 1 month (*p* = 0.93, Figure 1B).

### 3.2. Blood Chemistry and Biochemical Analysis

No significant changes or differences between groups were detected after two and 4 months for cholesterol, triglycerides (TG), HDL, LDL-c or fasting blood glucose for FVC vs. Placebo groups (all *p*’s > 0.05, Table 2, Table A3, Appendix A). Consumption of the meal replacements shake for an additional 4 weeks did not result in changes in plasma lipid measurements (Table 3, *p* > 0.05). However, the group consuming the meal replacements shake had significantly higher fasting blood glucose levels compared to the habitual breakfast group (101.2 ± 9.4 vs. 94.6 ± 5.3 mg/dL, *p* = 0.037, Table 3), despite a lack of difference prior to the consumption of the meal replacement shake (98.1 ± 8.6 vs. 98.3 ± 12.7 mg/dL, *p* = 0.79). The FVC supplementation with or without the meal replacement did not alter levels of oxidized LDL at two (*p* = 0.32), four (*p* = 0.94) or five months (*p* = 0.46, Table 2 and Table 3)

To determine immunomodulatory effect of the FVC supplementation, systemic cytokine and adipokine were measured. No treatment-induced changes were detected for MCP-1, TNF-α, IL-10. IL-1β, IL-6 and Leptin (Table 4 and Table 5). MIP-1β showed significant differences at 2 (*p* = 0.02) and 4 months (*p* = 0.04) after start of FVC supplementation, however, the level of MIP-1β tended to be different at baseline (*p* = 0.06). The GEE analysis further demonstrated both a timexgroup interaction that was trending but not significant (*p* = 0.089, Table A3, Appendix A). Levels of zonulin in stool and serum did not differ between groups at 2,4 or 5 months (*p* > 0.28, Table 4 and Table 5).

### 3.3. Fecal Short Chain Fatty Acids Analysis

Fecal SCFAs were measured using ion chromatography. Acetate was the most abundant SCFA detected and differed significantly between FVC and Placebo groups at baseline (*p* = 0.04, Table 6). GEE analysis of the SCFA identified butyrate as having a significant interaction between group and time (*p* = 0.043, Table A3, Appendix A) where butyrate was increased in the FVC group. No differences were detected with the meal replacement shake (*p* > 0.05, Table 7).

### 3.4. Fecal Microbiome

**Figure 2:**
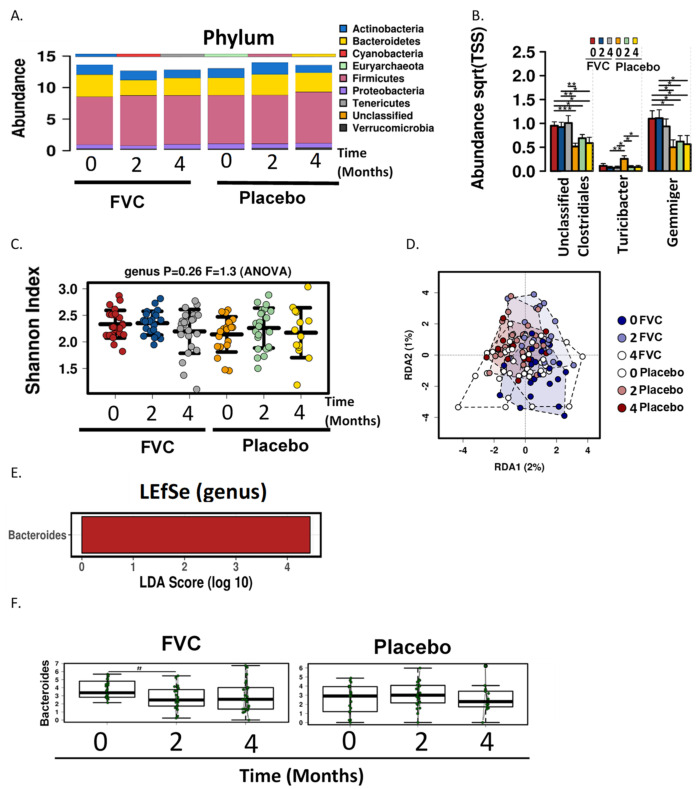
Microbiome abundance and diversity with FVC supplementation. (**A**) Fecal microbiota taxonomic abundance (phylum level) and (**B**) bar charts of abundance for the genera unclassified Clostridiales, Turicibacter and Gemminger for the FVC and placebo groups at baseline (0) and 2 and 4 months. No significant changes were observed at phylum level, but genus level difference existed between groups. * *p* ≤ 0.05, ** *p* ≤ 0.01, *** *p* ≤ 0.001. ANOVA. (**C**) Scatterplot showing differences in α-diversity at genus level determined by Shannon index of the fecal microbiome at baseline and months 2 and 4 for the FVC vs. placebo groups. No differences were detected. (**D**) Redundancy analysis (RDA) plot demonstrating β-diversity of the fecal microbiome at baseline and months 2 and 4 for FVC vs. placebo. No significant differences were detected. (**E**). Linear discriminant analysis (LDA) effects size (LEfSe) analysis shows differentially abundant genera with an LDA score *>*4 within the FVC group. (**F**) Box plots showing levels of Bacteroides for FVC and placebo groups at baseline, month 2 and 4. ^#^ *p* = 0.08, Mixed linear regression. (N = 27, FVC; N = 30, placebo) FVC, dried fruit and vegetable supplement.

Changes in fecal microbiome were determined by sequencing of the V4 region of the 16S rRNA gene. The most abundant phyla detected at baseline in both groups were Firmicutes (FVC, 74.5 ± 15.05%; Placebo, 77.02 ± 6.13%) and Bacteroidetes (FVC, 17.67 ± 10.61%; Placebo:12.8 ± 14.4%); followed by Actinobacteria (FVC, 3.6 ± 3.6%; Placebo, 6.9 ± 12.75%), Proteobacteria (FVC, 2.9 ± 10.45%; Placebo, 1.37 ± 2.45%) and Verrucomicrobia (FVC, 0.65 ± 1.1%; Placebo, 1.79 ± 4.95%). Additional phyla that were detected represented less than 0.2%. High inter-individual variability was present; the relative abundance for Firmicutes ranged from 34.4 to 99.7%. Analysis of fecal microbiome at baseline and 2 month and 4 months after initiation of the FVC intervention demonstrated no significant changes at phyla level between the groups (Figure 2A). Analysis of abundance at genus level did identify Turicibacter, Gemminger and an unclassified population of Clostridiales as being significantly different between the FVC group and placebo (Figure 2B, *p* < 0.05). However, these differences were consistent with group differences and did not result from supplementation. No treatment-induced changes were observed for α diversity (Shannon index, *p* = 0.26, Figure 2C) and redundancy analysis demonstrated that β diversity was also unchanged with supplementation (variance = 2.2, F = 0.82, *p* = 0.98, Figure 2D).

LEfSe analysis identified the family Bacteroidaceae and the genera Bacteroides to be enriched in the FVC group at baseline (LDA score = 4.44, Figure 2E). Mixed linear regression analysis demonstrated a reduction in Bacteroides between baseline and month 2 (*p* = 0.08, Figure 2F), with no reduction in the Placebo group during the same timeframe (*p* = 0.2, Figure 2F).

**Figure 3:**
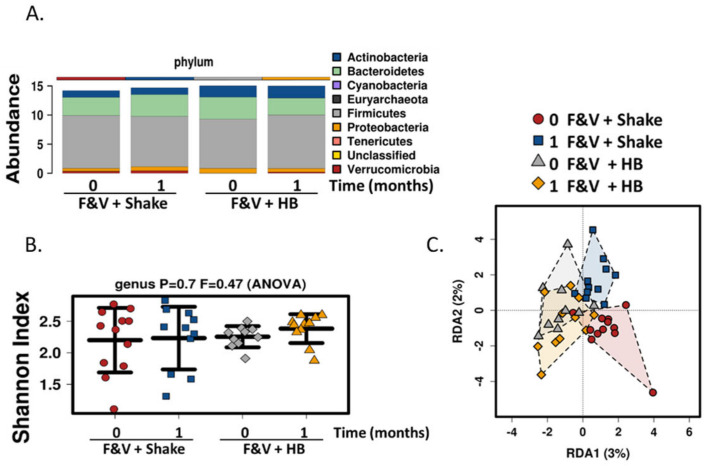
Microbiome abundance and diversity with consumption of the Complete^®^ high-fiber meal replacement drink (Shake) + FVC. (**A**) Fecal microbiota taxonomic abundance (phylum level) for the FVC+Shake vs. FVC+HB groups prior (0) and one month of supplementation (1). No significant differences were detected. (**B**) Scatterplot showing differences in α-diversity determined by Shannon index and (**C**) redundancy analysis (RDA) plot demonstrating β-diversity at genus level of the fecal microbiome prior (0) and after one month of Shake supplementation (1). No significant differences were detected. (*n* = 14, FVC + Shake; *n* = 12; FVC + HB) FVC, dried fruit and vegetable supplement; HB, habitual breakfast.

**Figure 4:**
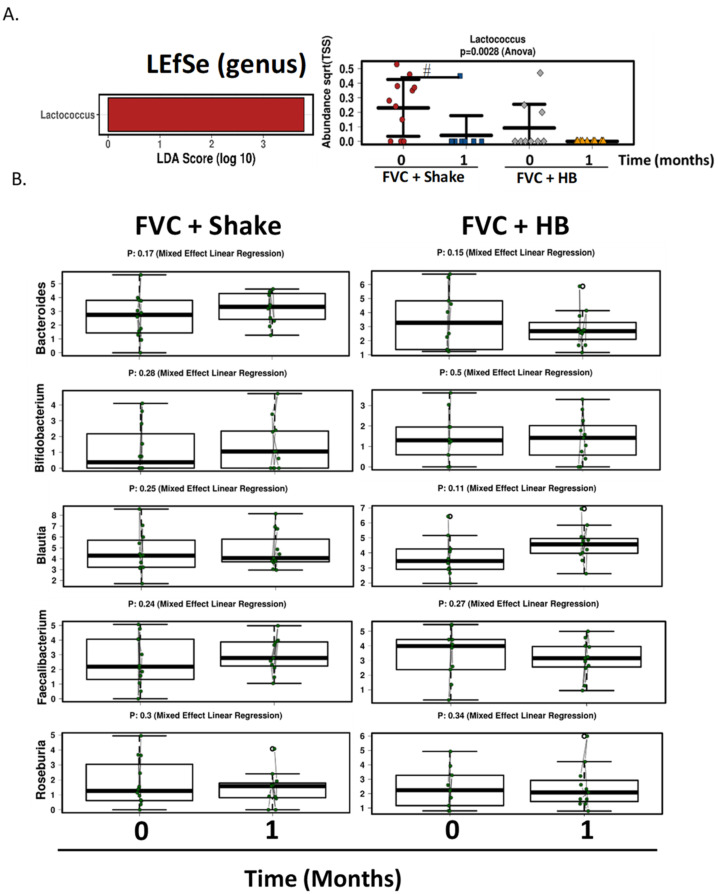
Genus level changes with Complete^®^ high-fiber meal replacement drink (Shake) + FVC. (**A**) Linear discriminant analysis (LDA) effects size (LEfSe) analysis shows enrichment for *Lactococcus* with an LDA score >3 within the FVC + Shake group (*Left panel*). Scatterplot of abundance of Table 1. month (0 vs. 1) of Shake consumption (*Right panel*). ^#^ *p* ≤ 0.05, ANOVA. (**B**) Box plots demonstrating the top significant genera (Bacteroides, Bifidobacterium, Blautia, Faecalibacterium, Roseburia) altered by Shake consumption for 1 month (mixed linear regression, *n* = 14, FVC + Shake; *n* = 12; FVC + HB) FVC, dried fruit and vegetable supplement; HB, habitual breakfast.

Consuming the high fiber shake as a meal replacement for one month in conjunction with the fruit and vegetable supplement did not result in significant changes at phylum level analysis. Firmicutes, Bacteroides and Actinobacteria remained the most abundant phyla (Figure 3A) and α-diversity measured by the Shannon diversity index was unchanged by the shake (*p* = 0.7, Figure 3B). Redundancy analysis showed no significant difference between groups (variance = 4.19, F = 0.89, *p* = 0.83), but there was a more dramatic non-significant shift in β-diversity when consuming the FVC+Shake for one month (Figure 3C). LEfse analysis further identified the genera Lactococus as being enriched in the FVC+Shake group at month 4 (LDA score = 3.78), but one month of the high fiber shake consumption the Lactococcus was significantly reduced this population (*p* = 0.0028, Figure 4A). Apart from Lactococcus, there were no significant changes induced by the shake. The shift in the RDA results from non-significant changes in the following genera: increases in Bacteroides (*p* = 0.17), Bifidobacterium (*p* = 0.28), Feacailibacterium (*p* = 0.24) and Roseburia (*p* = 0.3) and decrease in Blautia (*p* = 0.25) relative to the habitual diet group (Figure 4B).

## 4. Discussion

In the current study we evaluated the effect of a dried fruit and vegetable supplement with and without a high-fiber component on various parameters, including gut health, immune function, and supplementation-induced alterations in the intestinal microbiome in an obese, female population. The worldwide occurrence of obesity has dramatically increased and in women the prevalence has risen from 6.4% in 1975 to 14.9% in 2014 [27]. In addition to excess adiposity, obesity also creates a state of systemic slow grade inflammation that increases risk for diseases such as type 2 diabetes. The etiology of obesity is multifactorial, but over the last few years, it has become evident that the gut microbiome is altered in the obese state [28]. This is largely attributed to changes in diet that are concurrent with the incidence of obesity, as diet is a critical factor regulating the microbial community, diversity and structure [29,30,31,32].

The study participants were considered overweight/obese but were otherwise healthy with no metabolic dysfunction. Throughout the study period, participants followed their normal diet (~20% protein, ~20% fat and ~60% carbohydrates, data derived from a 7-day food diary) and lifestyle. Their fasting glucose levels were at the higher end of what is considered normal (<100 mg/dL).

Supplementation with the polyphenol rich dried fruit and vegetable capsules did not affect the measured metabolic parameters in the study population; no changes were detected for blood lipid and fasting glucose levels. Further evaluation of carbohydrate metabolism using an oral glucose tolerance test demonstrated a tendency towards improved glucose clearance with the dried fruit and vegetable supplement. Various studies have examined the association of a fruit and vegetable rich diet with risk of insulin resistance and type 2 diabetes, and polyphenols, secondary metabolites found in fruits and vegetables, have been shown to be associated with decreased risk for type 2 diabetes [33]. Polyphenol is suggested to inhibit α-amylase and α-glycosidase activities and also intestinal glucose transporters thereby reducing glucose absorption [34]. A recent meta-analysis of 18 studies found that diets rich in polyphenols, and particularly flavonoids play a role in the prevention of type2 diabetes [35]. In a placebo-controlled clinical study using a high-dose hyperinsulinemic-euglycemic clamp, Stull et al. also demonstrated that supplementation with whole blueberries improved insulin sensitivity without changing adiposity or inflammatory markers in obese, non-diabetic and insulin resistant participants [36].

Only 5–10% of total polyphenols are directly absorbed through the stomach and small intestine and therefore the majority of the ingested polyphenols reach the colon where they are extensively metabolized by bacteria [37]. In the current study, analysis of the gut bacteria using fecal samples revealed that the mean abundance of Firmicutes in the study cohort was 75.8%. This is consistent with a study by Chaves-Cagaja et al., who analyzed the gut microbiome of Mexican women that were of normal weight, obese or have been diagnosed with metabolic syndrome, and found that obese females had a relative abundance of 72.97% Firmicutes vs. 56.95% in normal weight females [38]. In the present study, genus level analysis revealed that the in FVC group the genera Gemmiger and Clostridiales (unclassified) were more abundant for the duration of the study. The abundance of Turicipbacter was higher in the placebo group at baseline but was reduced at months 2 and 4, with ending levels similar to that of the FVC group. The fruit and vegetable supplement did not significantly alter either α or β diversity of the microbial communities.

LEfse analysis identified the family Bacteriodaceae and genus Bacteroides as being enriched in the FVC group at baseline, but after 2 months a significant reduction was observed with the FVC supplementation. Bacteroides are anaerobic and bile resistant gram-negative rods [39] and have been positively correlated with long-term diets rich in animal protein and saturated fat [40]. Enrichment of Bacteroides vs. Prevotella was also associated with a diet that contains a low proportion of plant-based foods [41]. This genus has been found to be more abundant in populations with type 2 diabetes [42] and patients with celiac disease [43]. Various studies have studied polyphenols as inhibitors of microorganism growth and found that compounds found in black and green tea can inhibit the growth of pathogenic bacteria e.g., *Helicobacter pylori* [44]. Moreover, tea phenolics can significantly inhibit *Bacteroides spp*. growth, while commensal such as *Bifidobacterium spp*. and *Lactobacillus spp*. are less affected [45].

An increase in butyrate was observed with the polyphenol rich supplementation in the current study. Butyrate is a catabolic end-product of fermentation of dietary fiber and is preferentially used as an energy source by the gut mucosa [46]. Butyrate-producing bacterial species are found in the two most abundant families of Firmucutes; Ruminoccaceae and Lachnospiraceae. Within the Ruminoccaceae family Faecalibacterium prausnitzi has the metabolic capability of producing butyrate, while Eubacterium rectale and the related Roseburia species constitute the major butyrate-producers within the Lachnospiraceae family [47,48]. Butyrate has also been shown to play a role in glucose metabolism; Gao et al. demonstrated using a diet-induced obese murine model that butyrate supplementation can improve insulin sensitivity [49].

Polyphenols have been suggested to be a prebiotic, although this statement remains controversial. While it is recognized that polyphenols can modulate the microbial composition, evidence of prebiotic functions remains inconclusive [50]. In animal studies polyphenols such as catechins, anthocyanins and proanthocyanidins have been shown to increase what are considered as pro-health bacterial strains; Lactobacillus, Bifidobacterium, Akkermansia, Roseburia, and *Faecalibacterium spp* [50].

In addition to the polyphenol rich dried fruit and vegetable supplement, we also added fiber component in the form of a meal replacement drink, to determine its effect on the gut microbiome and health parameters. The drink contained various fiber types including plant cellulose, fructooligosaccharides, soy fiber, rice bran and pectin. There was a significant increase in fiber consumption in subjects consuming the meal replacement drink. Similar to the fruit and vegetable supplement, no changes were seen in general health, gut and immune parameters and also in α diversity gut microbial composition. Although β diversity was not significantly different, there was a slight shift in population composition with the inclusion of the high fiber smoothie; Lactococcus levels were significantly reduced and there were increases, albeit non-significant, in bacterial populations that are known to respond to prebiotics including Bifidobacterium, Faecalibacterium and Roseburia. Lactococcus is a lactic acid bacterium that is found in certain fermented foods. It is also found as part of the human gut microbiome and it associated with faster colonic transit time [51]. The polyphenol, resveratrol has also been shown to enhance the growth of *Lactococus lactis* [52]. Interestingly, the levels *of* Bacteroides were increased with the high fiber drink. This is consistent with various species within Bacteroides genus being able to metabolize complex carbohydrates [53]. Bacteroides and Bifidobacterium, and to a lesser extend Faecalibacterium and Roseburia have consistently been reported to potentially be protective against type 2 diabetes [54,55].

## 5. Conclusions

In conclusion, the results from the current study suggest that a dried fruit and vegetable supplement, rich in polyphenols, in combination with a high fiber meal replacement can alter the intestinal microbiota by inhibiting growth of specific bacteria while promoting the expansion of others. The increase in butyrate suggests there is an expansion of butyrate-producing species. During the same timeframe, there was also an improvement in glucose clearance suggesting that this combination of supplements can be used to improve glucose metabolism and possibly reduce the risk of insulin resistance and type2 diabetes.

## Figures and Tables

**Table 1:** Characteristics of subjects at baseline.

Parameter	FVC (N = 27)	Placebo (N = 30)	*p*-Value
Age, years	36.7 (±7.7)	35.1 (±8.0)	0.44
Weight, kg	81.1 (±14.3)	87.2 (±16.1)	0.13
Body mass index, kg/m^2^	30.7 (±4.4)	31.6 (±5.2)	0.49
Body Fat, %	36.5 (±4.8)	36.1 (±4.8)	0.77
Estradiol, pg/mL	64.4 (±77.8)	65.5 (±82.6)	0.96
Total Cholesterol, mg/dL	195.8 (±32.4)	181.2 (±34.8)	0.11
Triglycerides, mg/dL	101.2 (±45.7)	96.7 (±42.5)	0.70
Fasting glucose, mg/dL	97.67 (±13.9)	101.3 (±10.6)	0.26

Baseline data of 57 female participants. Values are mean ± SD. No significant differences were noted (*p* > 0.05).

**Table 2:** Fasting lipid and glucose levels for FVC vs. Placebo at baseline and month 2 and 4.

Parameter (mg/dL)	FVC (N = 27)				Placebo (N = 30)					
	Baseline	Month 2	Month 4	Baseline		Month 2	Months 4	p^1^	p^2^	p^3^
Cholesterol	195.8 ± 32.4	193.3 ± 29.1	196.2 ± 37.1	181.2 ± 34.8		182.0 ± 33.4	182.0 ± 27.1	0.11	0.20	0.11
TG	101.2 ± 45.7	100.3 ± 43.4	106 ± 54.2	93.7 ± 42.5		100.2 ± 41.0	97.1 ± 40.5	071	0.93	0.63
HDL	59.6 ± 14.6	57.9 ± 10.8	59.6 ± 13.0	56.8 ± 13.8		55.1 ± 11.9	56.0 ± 12.2	0.51	0.19	0.25
LDL-c	117.1 ± 32.5	117.7 ± 29.2	118.4 ± 32.8	109.5 ± 30.2		110.4 ± 29.0	110.5 ± 24.8	0.20	0.36	0.36
Glucose	97.7 ± 13.9	99.0 ± 11.4	98.6 ± 10.4	101.3 ± 10.6		101.3 ± 11.0	100.1 ± 9.9	0.12	0.52	0.28
Ox-LDL (U/L)	93.1 ± 54.8	102.4 ± 51.6	104.7 ± 53.4	76.6 ± 41.2		84.5 ± 46.0	95.5 ± 53.0	0.78	0.32	0.94

Values are mean ± SD. Non-parametric Wilcoxon test were used to compare differences between groups at baseline (p^1^), month 2 (p^2^) and month 4 (p^3^). No significance was detected.

**Table 3:** Fasting lipid and glucose levels for Shake vs. Habitual Breakfast at Month 5.

Parameter (mg/dL)	Shake (*n* = 14)	HB (*n* = 12)	*p*-Value
Cholesterol	197.5 ± 28.3	178.9 ± 34.8	0.14
TG	104.9 ± 49.1	97.3 ± 41.7	1
HDL	59.4 ± 14.8	56.3 ± 9.5	0.64
LDL-c	119.7 ± 28.6	104.9 ± 28.8	0.2
Glucose	101.2 ± 9.4	94.6 ± 5.3	**0.037**
OxLDL (U/L)	89.67 ± 58.6	73..9 ± 25.8	0.46

Values are mean ± SD. Significant differences at month 5 determined by non-parametric Wilcoxon test. Bold focus on significant data.

**Table 4:** Immune and intestinal health parameters for FVC vs. Placebo at baseline, month 2 and 4.

Parameter (pg/mL)	FVC (N = 27)			Placebo (*N* = 30)					
	Baseline	Month 2	Month 4	Baseline	Month 2	Month 4	p^1^	p^2^	p^3^
MCP-1	185.2 ± 92.7	186.6 ± 99.3	173.1 ± 87.6	200.6 ± 74.9	196.8 ± 83.7	199.7 ± 73.8	0.5	0.68	0.23
MIP-1β	26.1 ± 17.0	25.6 ± 15.8	34.7 ± 30.4	45.2 ± 49.8	44.1 ± 42.2	54.5 ± 41.9	0.06	**0.02**	**0.04**
TNF-α	6.8 ± 4.6	7.2 ± 5.0	11.7 ± 14.1	9.7 ± 9.7	10.4 ± 11.8	18.2 ± 22.3	0.25	0.62	0.67
IL-10	8.0 ± 6.6	7.9 ± 6.3	11.0 ± 13.4	8.3 ± 8.5	9.6 ± 10.0	14.8 ± 17.4	0.87	0.76	0.13
IL-1β	117.3 ± 269.4	167.1 ± 434	82.2 ± 182.9	26.8 ± 93.5	37.3 ± 131.6	13.7 ± 64.3	0.47	0.79	0.82
IL-6	9.1 ± 12.6	9.6 ± 11.6	11.1 ± 15.7	16.3 ± 44.3	8.2 ± 9.3	18.2 ± 29.5	0.81	0.52	0.46
Leptin	11925 ± 6667	12749 ± 8285	11961 ± 6373	11874 ± 7191	11026 ± 5457	11864 ± 6219	0.98	0.65	0.93
Zonulin (Stool, ng/mL)	185.1 ± 166.7	158.8 ± 68.6	184.7 ± 147.4	204.7 ± 241.9	205.9 ± 264.7	220.2 ± 239.9	0.94	0.7	0.93
Zonulin (serum, ng/mL)	61.2 ± 15.5	58.63 ± 14.9	56.8 ± 14.3	56.2 ± 14.8	57.6 ± 15.9	56.2 ± 15.8	0.2	0.97	0.97

Values are mean ± SD. Significance determined by non-parametric Wilcoxon test between groups at baseline (p^1^), month 2 (p^2^) and month 4 (p^3^). Bold focus on significant data.

**Table 5:** Cytokines and leptin for Shake vs. Habitual Breakfast.

Parameter (mg/dL)	Shake (*n* = 14)	HB (*n* = 12)	*p*-Value
MCP-1	218.7 ± 94.3	204 ± 96.8	0.65
MIP-1β	45.3 ± 27.0	40.88 ± 29.7	0.46
TNF-α	19.2 ± 22.7	15.14 ± 17.9	0.98
IL-10	16.5 ± 19.5	14.4 ± 18.0	0.87
IL-1β	8.5 ± 7.6	6.6 ± 6.8	0.35
IL-6	14.8 ± 14.6	13.2 ± 18.4	0.56
Leptin	16623 ± 9751	12554 ± 5576	0.49
Zonulin (stool, ng/mL)	350.4 ± 555.4	192.7 ± 165.3	0.90
Zonulin (serum, ng/mL)	54.5 ± 9.7	43.5 ± 20.2	0.28

Values are mean ± SD. Significance of Shake and Habitual Breakfast (HB) determined by non-parametric Wilcoxon test. No significance detected between groups.

**Table 6:** Fecal short chain fatty acid levels for FVC vs. Placebo.

Parameter (µmol/g)	FVC (N = 27)			Placebo (N = 30)					
	Baseline	Month 2	Month 4	Baseline	Month 2	Month 4	p^1^	p^2^	p^3^
Acetate	31.1 ± 13.0	33.2 ± 13.0	33.2 ± 13.0	36.3 ± 12.0	36.7 ± 14.0	31.8 ± 10.9	**0.04**	0.41	0.36
Propionate	13.0 ± 8.2	14.1 ± 6.9	14.1 ± 6.9	14.2 ± 6.5	14.1 ± 7.0	13.0 ± 6.4	0.21	0.87	0.81
Butyrate	13.9 ± 7.9	15.3 ± 8.7	15.3 ± 8.7	17.7 ± 10.3	17.8 ± 10.2	12.7 ± 6.7	0.14	0.70	0.99

Values are mean ± SD. Non-parametric Wilcoxon test were used to compare differences between groups at baseline (p^1^), month 2 (p^2^) and month 4 (p^3^). No significance was detected. Bold focus on significant data.

**Table 7:** Fecal short chain fatty acid concentration for FVC vs. Placebo groups.

Parameters (µmol/g)	Shake (*n* = 14)	HB (*n* = 12)	*p*-Value
Acetate	29.5 ± 9.1	35.6 ± 11.1	0.33
Propionate	10.9 ± 5.6	15.1 ± 4.8	0.12
Butyrate	11.6 ± 7.7	17.4 ± 9.4	0.18

Values are mean ± SD. Significance between Shake and HB determined by non-parametric Wilcoxon test.

## Data Availability

The data presented in this study are available on request from the corresponding author. The data are not publicly available according to consent by provided by study participants.

## Appendix A

**Table A1:** Nutrient analysis for FVC vs. Placebo at baseline and at month 4.

Nutrient (g/day)	FVC (N = 27)		Placebo (N = 30)			
	Baseline	Month 4	Baseline	Months 4	p^1^	p^3^
Protein	60.5 ± 14.6	61.1 ± 19.1	69.1 ± 18.6	71.0 ± 35.5	0.32	0.23
Fat	60.5 ± 20.6	63.4 ± 22.7	68.9 ± 19.9	72.5 ± 45.6	0.50	0.45
Carbohydrate	166.7 ± 44.8	184.4 ± 72.9	198.2 ± 58.0	194.8 ± 90.5	0.18	0.82
Total Fiber	12.5 ± 3.9	14.8 ± 5.9	13.5 ± 4.7	13.7 ± 6.5	0.63	0.67
Sugar	64.4 ± 31.6	74.2 ± 46.1	72.0 ± 35.6	73.8 ± 55.1	0.77	0.99
Kcal/day	1464 ± 370	1555 ± 497.3	1699 ± 429.7	1846 ± 1119	0.37	0.22

Values are mean ± SD. Non-parametric Wilcoxon test were used to compare differences between groups at baseline (p^1^) and month 4 (p^3^). No significant differences were detected.

**Table A2:** Nutrient analysis for Shake vs. Habitual Breakfast (HB).

Nutrient (g/day)		Shake (*n* = 14)	HB (*n* = 12)	*p*-Value
	Protein	79.2 ± 29.4	60.9 ± 17.9	0.08
	Fat	64.2 ± 24.1	60.0 ± 20.2	0.87
	Carbohydrate	200.8 ± 53.8	175.1 ± 68.6	0.56
	Total Fiber	22.3 ± 7.7	15.8 ± 5.0	**0.02**
	Sugar	87.5 ± 34.6	65.7 ± 30.4	0.32
	Kcal/day	1705 ± 452	1471 ± 463	0.39

Values are mean ± SD. Significance determined by non-parametric Wilcoxon test. Bold focus on significant data.

**Table A3:** GEE results for clinical parameters (FVC vs. Placebo).

Parameter	*β*1	*p*-Value	*β*2	*p*-Value	*β*3	*p*-Value
Estradiol	−7.20	0.731	−0.84	0.923	0.09	0.986
Glucose clearance	−22.60	**0.017**	−5.72	**0.021**	4.69	**0.094**
Fasting glucose	4.11	0.185	1.56	0.348	−1.34	0.270
Cholesterol	−13.47	0.111	−0.17	0.938	0.26	0.845
Triglyceride	−1.91	0.860	2.31	0.521	−1.11	0.606
HDL	−2.66	0.433	0.10	0.916	−0.11	0.840
LDL-c	−7.42	0.354	0.39	0.830	−0.08	0.947
Ox-LDL	11.74	0.3296	6.96	0.184	−2.18	0.503
Zonulin (stool)	−25.96	0.635	7.81	0.614	−3.95	0.701
Zonulin (serum)	5.32	0.183	1.26	0.4	−1.27	0.133
Leptin	1309.41	0.620	623.74	0.381	−614.95	0.305
IL-6	2.45	0.667	0.50	0.731	0.02	0.99
IL-1β	116.70	0.622	−17.72	0.183	7.48	0.288
IL-10	−1.65	0.486	−0.68	0.566	1.06	0.209
TNF-α	2.44	0.19	0.22	0.888	0.95	0.403
MIP1β	9.99	0.137	−7.76	0.204	9.93	**0.089**
MCP-1	15.04	0.497	−5.80	0.209	2.78	0.343
Acetate	19.95	0.12	6.97	0.187	−6.90	0.166
Propionate	7.84	0.441	2.69	0.314	−2.89	0.253
Butyrate	6.18	**0.053**	2.17	0.184	−2.31	**0.043**

Bold focus on significant data.
